# Antimicrobial Active Packaging Containing Nisin for Preservation of Products of Animal Origin: An Overview

**DOI:** 10.3390/foods11233820

**Published:** 2022-11-26

**Authors:** Elisabeta Elena Popa, Amalia Carmen Miteluț, Maria Râpă, Paul Alexandru Popescu, Mihaela Cristina Drăghici, Mihaela Geicu-Cristea, Mona Elena Popa

**Affiliations:** 1 Faculty of Biotechnology, University of Agronomic Sciences and Veterinary Medicine of Bucharest, 011464 Bucharest, Romania; 2 Faculty of Material Science and Engineering, University Politehnica of Bucharest, 060042 Bucharest, Romania

**Keywords:** nisin, natural preservative, packaging materials, animal origin products

## Abstract

The preservation of food represents one of the greatest challenges in the food industry. Active packaging materials are obtained through the incorporation of antimicrobial and/or antioxidant compounds in order to improve their functionality. Further, these materials are used for food packaging applications for shelf-life extension and fulfilling consumer demands for minimal processed foods with great quality and safety. The incorporation of antimicrobial peptides, such as nisin, has been studied lately, with a great interest applied to the food industry. Antimicrobials can be incorporated in various matrices such as nanofibers, nanoemulsions, nanoliposomes, or nanoparticles, which are further used for packaging. Despite the widespread application of nisin as an antimicrobial by directly incorporating it into various foods, the use of nisin by incorporating it into food packaging materials is researched at a much smaller scale. The researchers in this field are still in full development, being specific to the type of product studied. The purpose of this study was to present recent results obtained as a result of using nisin as an antimicrobial agent in food packaging materials, with a focus on applications on products of animal origin. The findings showed that nisin incorporated in packaging materials led to a significant reduction in the bacterial load (the total viable count or inoculated strains), maintained product attributes (physical, chemical, and sensorial), and prolonged their shelf-life.

## 1. Introduction

Nowadays, there is a great interest by consumers in minimally processed food products which were not subjected to severe thermal treatment, without chemical preservatives, but are still safe from a microbiological point of view and with a convenient shelf-life. Such products are hard to find, due to the fact that thermal and chemical treatments are often used for food preservation [1]; therefore, there is an increasing need to develop new techniques for potential microbial spoilage control and food shelf-life prolongation.

Food safety represents a prime concern for consumers, regulatory bodies, and manufacturers [2]. Microbial contamination and nutrient oxidation represent two main reasons for food spoilage [3], which still represents a great challenge worldwide, despite the continuous development of modern techniques in production [2]. One of these techniques is represented by active food packaging, an area of research where the packaging material fulfills functions beyond protection and containment. One category of active packaging has as its purpose in the reduction in food spoilage of the inhibition of the oxidative processes, leading to the diminishing of food waste by the addition in the packaging material of antioxidant and antimicrobial compounds capable to preserve product quality and prolong its shelf-life [4,5,6]. In this area, nanotechnology shows great potential [7].

Nisin belongs to the lantibiotic class of bacteriocins, is composed of 34 amino acids, and has a molecular mass of 3.5 kDa [8,9,10]. If its histidine (2-amino-3-(4-imidazolyl) propionic acid) or asparagine (2-amino-3-carbamoylpropanoic acid) is placed in position 27, then the bacteriocin is called nisin S or nisin Z. Nisin is a natural polypeptide obtained from some *Lactococcus lactis* strains [11,12,13], which presents antimicrobial activity against gram-positive bacteria [14,15,16], with low or no antimicrobial activity against gram-negative bacteria, yeasts or molds [17,18]. It is widely used as a food preservative [19] being recognized as safe (GRAS) [20,21,22,23] and approved by the EU and FDA [24,25]. According to Annex II of Regulation (EC) No 1333/2008, the maximum level of nisin (E 234 in the food additive list) permitted in foods is as follows: 10 mg/kg in other creams, unripened cheese (excluding products falling into category 16); 12.5 mg/kg in ripened cheese, processed cheese, and cheese products (excluding products falling into category 16); 6.25 mg/L or mg/kg in processed eggs and egg products, and 3 mg/L or mg/kg in desserts (excluding products covered in categories 1, 3, and 4) [26]. However, in 2017, the EFSA Panel on Food Additives and Nutrient Sources added to Food (ANS) proposed the extension of using nisin as a food additive in unripened cheese to a maximum level of 12 mg/kg and in heat-treated meat products to a maximum level of 25 mg/kg, concluding that these levels are not of safety concern [27].

The solubility and stability of nisin depends on their pH values, drastically increasing with the lowering of pH, being stable at pH 2.0, and almost insoluble in alkaline and neutral conditions. At a 2.0 value of pH, it can also be autoclaved for 15 min at 121 °C, without being inactivated [28].

Nisin has been studied for its incorporation into food packaging materials, but using it in its free unencapsulated form was associated with loss of activity due to degradation or deactivation. Therefore, the incorporation of nisin into films or as particles of edible polymers could protect it from protease action and also against environmental factors (temperature, pH, oxygen, etc.,) limiting its interaction with food components [29]. Nisin can be encapsulated without the use of nanotechnology by various techniques, such as liposome entrapment (using liposomes as micro and nanocarriers), coacervation (where the entrapment of the antimicrobials within wall materials can occur by adding non-solvent substances or electrolytes or by altering temperature or pH, the processing factors), emulsification (obtaining micro or nanoemulsions by various techniques, such as phase inversion, microfluidization, high-pressure homogenization, or ultrasonication), spray-drying (having great advantages, such as cost-effectiveness, the usage of already available equipment, or the obtaining of dried and stable capsules in a single step process), or vibrating technology (producing controlled size polymer beads) [30], with the aim of being used directly into foods or added on/incorporated in packaging materials.

The purpose of this study was to present the results obtained as a result of using nisin as an antimicrobial agent in food packaging materials with specific applications on meat and dairy products.

## 2. Background Theory

### 2.1. Nisin Mechanism of Action

The mechanism of action of nisin, as described by various studies, refers to its ability to disrupt the membrane integrity [31,32]. Therefore, the following steps take place: (i) nisin binds to specific cell walls (of target cells) to inhibit their biosynthesis [32], (ii) pore formation disrupts the pH equilibrium and proton motive force [33], (iii) leakage of intracellular constituents [34], and (iv) cell death or inhibition of its activity [35]. Furthermore, Khan and Oh (2016) [32] reported that nisin is more effective against spores compared to vegetative forms of microorganisms due to its sporostatic potential, which is greater than its sporicidal activity.

**Figure 1:**
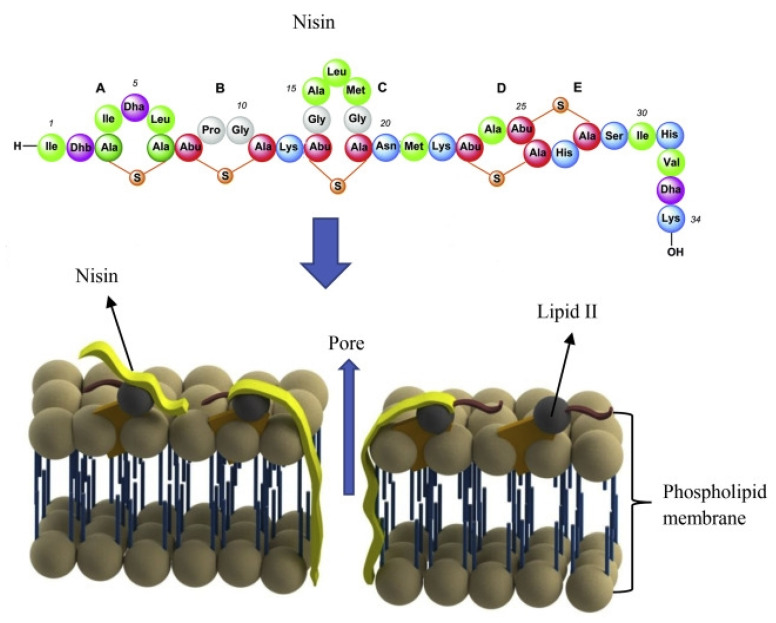
Antimicrobial mechanism of nisin action to the killing of bacteria. Reproduced from Bahrami et al. (2019) [36] with permission from Elsevier.

The antimicrobial mechanism of nisin in food packaging is revealed in Figure 1. Accordingly, the disruption of cell membranes is the first step to the killing of bacteria, followed by the infiltration of nisin to the precursor material for the biosynthesis of the bacterial wall (named lipid II). In this way, nisin acts against the growth of the peptidoglycan network. A pore in the cell membrane appears when the N-terminal part of nisin joins with the carbohydrate-pyrophosphate part of lipid II [36]. To be effective against the inhibition of microorganisms, nisin must be in high concentration to exert a high release. The slow release of bacteriocin can be effective when the initial bacterial count of the packed food is low [37].

### 2.2. Incorporation of Nisin in Packaging Materials

Packaging’s main role is to protect food products from external factors during all steps of production. Active packaging represents a modern technology used for packing food products, where different biological substances with antimicrobial/antioxidant activities can be incorporated into the packaging [38] to enhance the products’ functionality and prolong their shelf-life. According to Regulation (EC) No 1935/2004 of the European Parliament and of the Council (of 27 October 2004 on materials and articles intended to come into contact with food and repealing Directives 80/590/EEC and 89/109/EEC, 2004 within the general requirements for food contact materials), “Materials and articles, including active and intelligent materials and articles, shall be manufactured in compliance with good manufacturing practice so that, under normal or foreseeable conditions of use, they do not transfer their constituents to food in quantities which could endanger human health or bring about an unacceptable change in the composition of the food, or bring about a deterioration in the organoleptic characteristics thereof” [39]. It is of high importance that the aspect of food contamination due to the migration of components from food packaging materials is addressed so, in this respect, the European Union implemented a legal document that lists the substances that can be used for packaging materials development [40]. This document (Commission Regulation (EU) No 10/2011 of 14 January 2011 on Plastic Materials and Articles Intended to Come into Contact with Food, 2011) also presents the conditions for migration testing and limits for different added substances [41].

In the food industry, the main source of human exposure to nanoparticles is when they are intentionally added to the food product. The migration of nanoparticles from the packaging to the food product is a source of indirect exposure [42]. This process can occur due to processes such as dissolution, diffusion, or abrasion of the packaging material, being also dependent on different factors such as food properties, environmental conditions, packaging materials properties, nanoparticles properties (molecular weight, particle size, and solubility), and also by the interaction between the polymer matrix and nanoparticles [43]. Furthermore, this process could lead to toxicity [44], mainly due to nanoparticle properties such as non-biodegradability, non-dissolvability, and persistency. Workers in companies producing nanocomposites are directly exposed to nanoparticles, a fact that can cause them health problems either as a result of inhalation or direct contact with the skin. Maisanaba et al. (2015) [45] showed that nanoparticles can cause inflammation and oxidative reactions, a consequence of their ability to cross the cell barrier. However, more studies are needed on the extent to which nanoparticles migrate from packaging to the food product, how they behave after entering the body, and how they are absorbed by different organs [46]. The possible health risks associated with the ingestion of nanoparticles must be correlated with various parameters, such as size, toxicity, rate of migration, and the extent to which the human body absorbs the nanoparticles [47]. For example, Han et al. (2012) [48] stated that small-sized nanoparticles are more dangerous to human health than large-sized ones, as they can be distributed in the body and organs more easily. Moreover, the smaller the nanoparticles, the higher their absorption rate.

However, nisin has been used widely in the food industry, being approved by the European Food Safety Authority, US Food and Drug Administration, and also by the Food Standards Australia New Zealand [49]. Until now, there are no reported studied about the toxicity of nisin nanomaterials used for food applications. Furthermore, studies have been made on the migration properties of nisin-incorporated packaging materials. For example, Hanušová et al. (2010) [50] studied nisin migration from polyvinyl dichloride lacquer coating into acidified physiological solution, determining a maximum level of nisin migration of approximately 800 ± 7 IU/dm^2^. Remedio et al. (2019) [18] developed chitosan films incorporated with nisin. Nisin was released at an accelerated from the developed film (80% of antimicrobial released in the first 15 min of testing), the diffusion coefficient was 1.234–1.347 × 10^−13^ m^2^/s, and this fact was associated with the high molecular mass of nisin (3354 g/mol).

A composite film containing nisin was studied for its migration properties by Reichenberg et al. (2015) [51] in deionized water. After 24 h, it was observed that the final concentration of different samples is between 2–6 µg/mL, which corresponds to 0.6–2.0 wt% of total released nisin content.

Chang et al. (2021) [52] developed PLA-based films by direct incorporation of nisin as an antimicrobial agent to be used as food contact materials. Further, they researched the migration properties of the developed materials and found that the overall migration values were very low in comparison with the limit set by the European Commission, which is 10 µg/dm^2^, proving the safety of the tested films.

Nisin incorporated into sugarcane bagasse nanocellulose by solvent casting allowed the use of such hybrid film as a liner of low-density polyethylene (LDPE) plastic packaging for ready-to-eat ham. After 7 days of storage at 4 °C, it was reported the inhibition of the growth of *Listeria monocytogenes,* according to Regulation (EC) (2073/2005) [53].

Furthermore, nisin is considered a preservative within the food industry, and is considered safe for human usage in 1969, according to the World Health Organization (WHO). The stability and antimicrobial activity of nisin are the main drawbacks of using this bacteriocin in food applications. Nanoparticles are recognized for their ability to improve the controlled release, stability, and bioactive properties of nisin [54,55,56].

The stability and bioactivity of nisin loaded onto mesoporous silica nanoparticles (MSNs) were investigated against *Staphylococcus aureus* and by in vitro cytotoxicity [54]. The results showed that both free nisin and nisin loaded onto MSNs have no significant toxic effects on the mouse fibroblast L929 cells.

Nisin-loaded poly-γ-glutamic acid (γ-PGA)/poly-l-lysine nanoparticles (PLL) showed good stability, proved by zeta potential values above 30 mV for the nisin concentrations of 0, 3.0 and 4.0 mg/mL [56]. Further, the stability of nisin led to enhanced antibacterial activity of nisin-loaded y-PGA/PLL nanoparticles against Staphylococcal enterotoxin A for up to 14 days at 4 °C and 7 days at 25 °C. In another paper, the testing of synthesized nisin-silver nanoparticles as antimicrobials against biofilm-forming pathogens was reported [57]. The cytotoxicity of nisin-silver nanoparticles performed on the human skin fibroblasts, and a human kidney epithelium cell line proved that after 24 h of incubation, the human cells were unaffected by the concentration of nisin at 128 μg/mL. Additionally, the nisin-silver nanoparticles are considered an efficient antimicrobial agent with low toxicity.

Wang et al. (2021) [58] studied the cytotoxicity of some antibacterial films (obtained by adding nisin and *Perilla* essential oil to N-succinyl chitosan). The results showed a lower lactate dehydrogenase release rate for all tested samples compared to the control group, demonstrating that tested samples presented no cytotoxicity.

The incorporation of nisin into antimicrobial food packaging involves either direct introduction into food, food packaging materials, or edible food packaging. The kinetic release of nisin from food packaging materials is governed by Equation (1) [59]:

(1) $$\frac{M_{t}}{M_{\infty }}=1-\frac{8}{\pi^{2}}\sum_{n=0}^{\infty }\frac{1}{{\left(2n+1\right)}^{2}}\exp\{-D{\left(2n+1\right)}^{2}\pi^{2}t/l^{2}\}$$

where *M_t_* and *M_∞_* are the mass of nisin released from the film at time *t* and at infinite time, respectively, *D* is the diffusion coefficient, and *l* is the film thickness.

Generally, the following steps occur at the releasing of nisin from food packaging: (i) the diffusion of water molecules from food into the packaging material, (ii) the relaxing of packaging components, (iii) an antimicrobial agent is diffused through the swelled packaging, and (iv) the delivery of the antibacterial agent from the packaging to the microbial surface.

Active packaging materials can inhibit the microbial load of food directly on its surface, where, in fact, contamination/spoilage occurs; therefore, the quantity of antimicrobial substances necessary for ensuring food safety is drastically decreased. Therefore, appropriate contact between the food product and the developed active packaging material must be ensured for a good diffusion of the incorporated antimicrobial peptide to diffuse and exert its action on spoilage microorganisms that could be present on the surface of the product [31].

Direct introduction of nisin into food caused its adsorption having a consequence on the missing antimicrobial activity. Therefore, Gharsallaoui et al. (2016) [60] proposed the incorporation of nisin into polymer matrices, which allows its controlled release at the food surface.

By using the processing of antimicrobial food packaging by electrospinning, the limitation of nisin as its agglomeration by other technologies can be removed, since the nanofibers are fabricated using solutions or melted polymers. Therefore, different nisin concentrations were introduced into a poly(ε-caprolactone) (PCL) solution to obtain active packaging with antibacterial activity against *Escherichia coli* and *Staphylococcus aureus*. It was reported that the addition of nisin lead to increased tensile strength, together with the decrease in elongation at break, explained by the action of nisin particles as a bulky barrier to reducing the macromolecular chains movement of PCL [61]. The same finding was observed by Yang et al. (2020) [53], when increasing the concentration of nisin at 0, 640, 1280, 1920, 2560, and 3200 mg/L into cellulose nanofibrils (CNFs) prepared from sugarcane bagasse. However, the antibacterial activity increased with the loading of nisin.

In order to overcome the loss of nisin antibacterial activity over time, Huang et al. (2020) [62] proposed the covalent immobilization of nisin on poly(lactic) (PLA) film by using plasma treatment, which produces carboxylic acid groups. The total viable count (TVC), *Pseudomonas* spp., lactic acid bacteria (LAB), total volatile basic nitrogen (TVB-N), drip loss, pH, and color of the beef were determined. After 15 days of cold storage, the TVC value was 6.83 ± 0.10 log CFU/g in the case of nisin−g−PLA film, which is under the permissible limit of the TVC value in fresh meat recommended by the International Commission on Microbiological Specifications for Foods (ICMFS). Other physical-chemical parameters decreased compared with the control, suggesting the effective role of nisin to prolong the shelf-life of as fresh beef.

The recognized techniques for edible food packaging containing nisin involved coating [63,64] and casting [37,53,59]. A composition based on nisin, zein, starch, tapioca, natamycin, and glycerol was made for preparing the edible film with a thickness of 0.162 ± 0.002 mm by the casting method. The improved mechanical properties, in terms of the decreased elongation at the break, barrier property, and film dissolving in water without reducing the antimicrobial activity of film were reported [65].

A comparative study about the nisin activity of films fabricated by hot-pressing and casting methods performed by Dawson et al. (2003) [66] showed that greater antimicrobial activity was retained in the case of casting film. This behavior is explained by the high temperature involved in hot-pressing technology, which diminishes the antibacterial activity of nisin. When comparing different film materials containing nisin obtained by the casting method, it was reported that the low pH of the solution better retains the antibacterial activity of nisin [66].

## 3. Application and Effects of Nisin Incorporated Packaging Materials

### 3.1. Antimicrobial Activity of Materials Containing Nisin

Antimicrobial peptides, such as bacteriocins, have been used in food preservation for many years, containing compounds generally recognized as safe (GRAS) [67]. Nisin is a heat-stable, nontoxic [68], harmless bacteriocin which is commercially available to be used in various food products [31], such as meat and meat products, dairy products, vegetables, juice, baked goods, and canned goods [36,69], which has proven its antimicrobial efficiency, inhibiting the growth of various microorganisms [70]. However, some challenges that can limit the use of nisin should be addressed, such as sensitivity to stresses found in the environment, uncontrolled antibacterial efficiency during storage of food products, susceptibility to proteolysis, and unwanted interactions with food components. To control such challenges, a sustained release of nisin should be performed during product shelf-life by obtaining efficient delivery systems [32], such as active packaging obtained by various methods within the nanotechnology area (e.g., casting, encapsulation, and electrospinning).

Various studies reported nisin having a strong antimicrobial activity, especially against gram-positive bacteria such as *Enterococcus*, *Staphylococcus*, *Bacillus cereus*, *Lactobacillus*, *Mycobacterium*, *Leuconostoc*, *Listeria monocytogenes*, *Clostridium botulinum*, *Clostridium sporogenes*, *Micrococcus,* and *Pediococcus* [31,32,34]. Less inhibitory activity was acknowledged for gram-negative bacteria, yeasts, and molds [34]. Therefore, materials containing nisin can be used in combination with chelators and/or other antimicrobials (the hurdle approach) to enhance antimicrobial activity against gram-negative bacteria.

To avoid the nisin resistance to the gram-negative bacteria, due to its outer membranes, its combination with other antimicrobials and chelating agents, polycationic acid, or organic acids [71,72,73] was proposed. By microencapsulation of nisin in the presence of sodium alginate concentration (2% *w*/*v*) and guar gum concentration (0.4% *w*/*v*), the encapsulation efficiency of nisin was 36.65% [74]. Although this parameter is not very high, it is expected that the controlled release and antimicrobial activity of nisin to be increased.

Wang et al. (2017) [58] studied the combined effect of nisin, potassium sorbate, and bacteriophage on fresh chilled pork contaminated with *Salmonella*. The combining treatment of the three antimicrobials significantly decreased *Salmonella* counts and inhibited bacterial growth. Furthermore, the organoleptic properties of samples were improved and the shelf-life was extended up to 14 days.

In contrast to the nisin alone, the antimicrobial potential of nisin increased after conjugating with biogenic metallic nanoparticles. Thus, the testing of conjugated nisin with silver nanoparticles obtained from extracellular cell-free extracts of *Phanerochaete chrysosporium* demonstrated antimicrobial activity against *Staphylococcus aureus* and *Escherichia coli* [75].

As chelating agents, different studies used food powders such as calcined diatomaceous earth, synthetic calcium silicate, corn starch [76], disodium ethylenediamine tetra acetic acid (EDTA), and sodium hexametaphosphate (HMP) [73]. When nisin–EDTA or nisin–HMP are immobilized together with calcium alginate gel at 10 °C, the reduction in *Escherichia coli* O157:H7 growth in ground beef was enhanced [73]. This behavior was explained due to the lowering effect of calcium alginate gel at the tested temperature rather than nisin.

Morsy et al. (2018) [77] determined the synergistic effects of nisin, lysozyme, EDTA nanoparticles, or ZnO nanoparticles on *Escherichia coli* O157:H7, *Listeria monocytogenes,* and *Bacillus cereus* as foodborne pathogens related to minced beef, during storage at refrigeration temperatures. The most synergistic combinations were lysozyme-nisin-ZnO and lysozyme-nisin-EDTA-ZnO, which presented inhibitory effects on all three tested bacterial strains.

In a study performed by Chang et al. (2021) [52], it was demonstrated that the metal ion chelating agent EDTA–nisin improved the antimicrobial activity of PLA-chitosan films produced by extrusion on a casting laminating machine designed for packaging of fish fillets at 25 °C or 4 °C. The EDTA acts to destroy the stable cell structure of gram-negative bacteria by binding the metallic cations to nisin, which creates pores on the cell surface.

The synergistic action of nisin, ascorbic acid, and EDTA in synthetic solutions with high antimicrobial activity against *Salmonella enteritidis* was studied by using the response surface methodology method (RSM) [72]. A reduction number of 3.41 log colony forming units (CFU) was achieved at the optimal concentrations of nisin, ascorbic acid, and EDTA of 500 ppm, 1515 ppm, and 250 ppm, respectively, demonstrating the higher antimicrobial activity of the three antimicrobial components as compared with only two of the tested antimicrobial agents [72].

Therefore, such antimicrobial agents are ideal for developing antimicrobial films [78] as active packaging, which can be successfully used in increasing the shelf-life of food products [18], maintaining their quality and safety. In this respect, Table 1 presents the most recently developed packaging materials containing nisin and their antimicrobial effect.

Hurdle technology is used for food preservation and it was developed as a new concept for obtaining food products with great characteristics such as enhanced safety, nutritional compounds, or taste [103]. Therefore, the application of extra hurdles in food processing for microbial growth control could lead to safer products with a prolonged shelf-life, including raw or ready-to-eat food products [104].

Zhang et al. (2021) [78] studied the combined effect of nisin, oregano essential oil, and modified atmosphere packaging on the shelf-life of grass carp. The addition of oregano essential oil and nisin alone and in combination led to a significant increase in grass carp shelf-life, inhibiting the microbial load on fish fillets. Another study on the combination of nisin with modified atmosphere packaging was performed by Naas et al. (2013) [105], which evaluated the development of *Listeria monocytogenes* in ready-to-eat turkey bologna. The result showed that nisin action reduced bacterial load by 1.5 to 2.0 log cycles and the presence of 100% CO_2_ within the package prevented the growth of bacteria during 42 days of storage; their combination being an efficient way to increase the shelf-life of the tested product.

The combining effect of 1000 µg/g nisin and 50 mM EDTA solutions on the packaging of Turkish-type meatballs with inoculated solutions at 4 °C in a vacuum or modified atmosphere packaging (MAP) had, as a result, the reduction in the viability of *Salmonella enteritidis* [106].

The inactivation of *Listeria innocua* was researched by Costello et al. (2021) [23] through the combining effects of nisin and cold atmospheric plasma treatments. It was shown that when nisin was applied before cold atmospheric plasma treatment, its combination was more efficient than the treatments applied alone, proving that an additional hurdle can improve the efficiency of nisin in food preservation.

Economou et al. (2009) [107] tested the efficiency of nisin–EDTA treatments combined with modified atmosphere packaging on the extension of the shelf-life of chicken meat. The results showed an increasing shelf-life of samples depending on the amount of nisin/EDTA added; the longest period being obtained by a combination of modified atmosphere packaging and treatment of 500 IU/g of nisin plus 50 mM of EDTA (shelf-life prolongation by 13–14 days in refrigerated conditions).

Martillanes et al. (2021) [108] developed an antimicrobial film based on chitosan and nisin and used it in combination with high hydrostatic pressure processing for the reduction in *Listeria monocytogenes* previously inoculated on the surface of sliced dry-cured Iberian ham. The results of the study showed that this combination was the most efficient in bacteria inhibition in comparison with the treatments alone, presenting a reduction in its count of 6.1 CFU/g. The combination of high-pressure processing and nisin was also studied by García et al. (2022) [24] to determine the microbial quality of micellar casein concentrates in liquid form. The results showed low inhibition when using a high-pressure processing treatment at 300 Mpa alone, but when nisin was added the results improved. However, the greatest effect was achieved at a higher pressure of 450 Mpa combined with nisin, where an efficient microbial growth control was obtained without affecting the properties of micellar casein concentrates.

Another technology that was used by Costello et al. (2021) [109] to enhance nisin antimicrobial activity is ultrasound. It has been demonstrated that ultrasound alone presented efficiency against *Escherichia coli* at 500 kHz, whereas no effect was observed on *Listeria innocua,* no matter the frequency used or nisin presence. However, the addition of nisin before ultrasound treatment led to enhanced inhibition of *Escherichia coli*, concluding that ultrasound could be an effective hurdle on nisin inhibitory properties against gram-negative bacteria.

Nisin combined with hurdle technology was also studied by Han et al. (2021) [110] on ready-to-eat shrimp. First, the shrimps were boiled, dried, nisin treated, seasoned, and roasted and then vacuum packed, sterilized, and stored at room temperature. The microbiological analysis showed that the combination of nisin with other hurdles led to a significant bacterial load decrease compared to the control sample.

Therefore, as presented above, additional hurdles applied in combination with nisin can enhance not only its antimicrobial activity on gram-positive and gram-negative bacteria, but also could lead to shelf-life prolongation of food products of animal origin.

### 3.2. Effects of Materials Containing Nisin on Meat and Dairy Products

Meat and meat products represent good sources of high-quality protein containing excellent amino acid ratios and high bioavailability, which could also provide the human body with other beneficial elements such as magnesium, selenium, potassium, iron, and vitamins, yet their high nutritional value makes preservation difficult [111]. The main causes of degradation are lipid oxidation and microbial spoilage [112]. Therefore, there is an increasing need to develop solutions for meat and meat products preservation, one of them being active packaging and the incorporation of active or antimicrobial compounds which leads to shelf-life extension [113].

Kaewprachu et al. (2018) [114] investigated if gelatin-based films incorporated with nisin and catechin were capable to prolong minced pork meat shelf-life. The results showed that the developed films retarded lipid oxidation and microbial growth; the time in which the meat reached a total viable count of 10^7^ CFU.g^−1^ was prolonged from 1 to 4 days.

The combination of coatings based on nisin/gallic acid/chitosan with high oxygen-modified atmosphere packaging on fresh pork loin was tested by Cao et al. (2019) [115], in order to determine their effect on preservation during cold storage. The results showed that using a coating formed by chitosan, nisin, and gallic acid was the best solution because it preserved better product color, had lower lipid and protein oxidation, had the lowest TBARS (thiobarbituric acid reactive substances) value, and presented synergistic bactericidal effects.

Active packaging based on nisin, EDTA, and PBAT/TPS (poly(butylene adipate terephthalate)/thermoplastic starch blends) was developed and tested on pork meat by Leelaphiwat et al. (2022) [116]. The study showed that PBAT/TPS films that contained EDTA and nisin efficiently inhibited lipid degradation in pork tissues. The microbial growth was also inhibited, a fact that led to the stabilization of meat redness and delayed discoloration, keeping the quality of tested packed pork meat.

Liu et al. (2020) [117] prepared a nanoemulsion incorporated with a blend of nisin, star anise essential oil, and polylysine which was used as a coating for Yao meat. The coating had no effect on sample moisture content for 20 days, maintaining good color and odor. Sample shelf-life was extended from 8 to 16 days.

Correa et al. (2017) [118] determined the effect of nisin adsorption on PHB/PCL (polyhydroxybutyrate/polycaprolactone) films, showing that the optimal parameters for this process were 4000 IU cm^3^, at a temperature of 40 °C and 10 min. The authors showed that the developed films were effective against *Lactobacillus plantarum* CRL691 inoculated on sliced ham, prolonging its shelf-life.

The effect of HPP (high-pressure processing) in combination with chitosan-based films containing nisin and/or rice bran on extract dry-cured Iberian ham inoculated with *Listeria monocytogenes* was evaluated by Martillanes et al. (2021) [108]. The results showed that the combination of HPP with the developed films reduced the population of *Listeria monocytogenes* by 6 log CFU/g.

Cellulose nanofibrils/nisin nanohybrid films were developed and tested on ready-to-eat ham by Yang et al. (2020) [53]. It resulted that the film completely inhibited *Listeria monocytogenes* during storage for 7 days at 4 °C.

Batpho et al. (2017) [119] developed antimicrobial collagen casting impregnated with nisin and applied it to Vienna sausage. The results showed that the developed antimicrobial casing extended the shelf-life of the sausage for at least 90 days at 4 °C and 49 days at 10 °C.

Pattanayaiying et al. (2015) [120] developed pullulan films containing lauric arginate (LAE) and nisin Z, alone or in combination, and investigated their effect on controlling foodborne pathogens in raw turkey breast slices. The study showed that *Salmonella typhimurium* and *Salmonella enteritidis* load was reduced during the testing period. Additionally, the film containing LAE and nisin effectively reduced *Staphylococcus aureus* and *Listeria monocytogenes* Scott A inoculated on ham surfaces during cold storage.

Morgan et al. (2022) [121] developed two coatings containing nisin (one of them was added with calcium chloride) and coated two types of materials used as substrates. Further, the obtained materials were tested on turkey bologna, and both developed coatings inhibited *Listeria monocytogenes* for the entire period of the turkey bologna antimicrobial package testing, being significantly different when compared to the control samples.

Morsy et al. (2018) [77] applied combinations of lysozyme 500 U/mL (L), nisin 1000 IU/mL (N), EDTA nanoparticles 5 mM (E), and ZnO nanoparticles 12 mM (Z) as a challenge test on minced beef. The greatest synergistic antimicrobial effect was demonstrated by LNZ and LNEZ, which inhibited effectively *Escherichia coli* O157:H7, *Listeria monocytogenes,* and *Bacillus cereus*, reducing the microbial load between approximately 2–4 log CFU cm^2^ in packaged minced beef during storage al 4 °C for 15 days.

Nanocoatings based on bacterial cellulose nanocrystals and nisin-loaded bacterial cellulose nanocrystals were applied on beef by spraying by Gedarawatte et al. (2022) [122]. No alteration of sensorial and physicochemical properties was observed when using these nanocoatings and the bacterial growth was reduced when using nisin, compared to the bacterial cellulose nanocrystals alone.

Edible nanoparticles were prepared using nisin, rosemary essential oil, and *Lycium barbarum* polysaccharides by Lin et al. (2022) [123] and then applied to beef. The preservation effect was favorable, without affecting beef color or texture.

Guohua et al. (2016) [124] determined the effect of chitosan combined with nisin in different concentrations on the quality of yellow croaker, during storage at 4 °C for 8 days. The study determined that the samples treated with nisin presented better quality parameters, such as moisture loss control, inhibition of spoilage, reduction in TVB-N (total volatile basic nitrogen), controlling total viable count growth, and maintaining color and sensory acceptability.

A combination of the coating formed from gel dispersion of seer fish incorporated with nisin and gamma irradiation was investigated by Kakatkar et al. (2017) [125] when applied to seer fish steaks. The study showed that the tested combination led to the shelf-life prolonging of samples from 7 days to 34–42 days during cold storage.

Meral et al. (2019) [126] developed nisin and curcumin nanomats, which were further applied as coatings on fish fillets. The control samples presented acceptable sensory attributes by the fourth day of storage, while the coated samples presented acceptable sensory attributes by the tenth day of storage. The shelf-life of the coated samples was extended by 12 days.

Oner et al. (2021) [127] combined nisin-loaded PVA-based nanofibers with poly(ethylene) (PE) in order to obtain new packaging material. The obtained material delayed TMB (total mesophilic bacteria) and LAB (lactic acid bacteria) development in fish fillets by 31% and 38%, respectively. The samples coated with the obtained material also presented better sensorial attributes compared to the control samples.

Pattanayaiying et al. (2019) [128] developed a thermoplastic starch/PBAT film, which was coated using gelatin with lauric arginate alone or in combination with nisin. Both films were then applied on bigeye snapper and tiger prawn slices during long-term (28 days), refrigerated (4 °C; chilled), and frozen (−20 °C) storage for up to 90 days. Both films reduced *Salmonella Typhimurium* ATCC 14,028 and *Vibrio parahaemolyticus* after 28 days at 4 °C, and also *Salmonella Typhimurium* after 60 days at −20 °C. *Vibrio parahaemolyticus* was also reduced when the coating containing nisin was used on frozen samples after 14 days (bigeye snapper) and 21 days (tiger prawn), while the coating without nisin reduced *V. parahaemolyticus* on both frozen seafood slices after 28 days.

The effect of chitosan/sodium alginate/nisin preservatives on the quality of *Penaeus vannamei* shrimp during cold storage was investigated by Cen et al. (2021) [129]. The results showed the treated samples presented lower pH, TVB-N, TVC (total viable counts), and freeness values compared to untreated samples. Additionally, the tested preservatives decreased the predominant microbial load significantly and led to a longer shelf-life.

Nisin and EDTA were added to the chitosan-PLA composite film by Chang et al. (2021) [52] and applied to fish fillets which led to a significant reduction in coliform, mesophile, the spoilage of bacteria loads, and of the total volatile basic nitrogen content in fish during storage at 25 °C and 4 °C.

Dairy products also represent a great group of food products with an extensive variety and geographic distribution [130], but with limited shelf-life due to spoilage microorganisms’ growth [131], which degrade milk components causing an unsatisfactory quality of the product [132]. Therefore, there is an increasing need to protect and prolong the shelf-life of such products. Lately, scientific studies focused on using active packaging to achieve this aspect.

Meister Meira et al. (2016) [85] developed some nanocomposite films based on starch/halloysite/nisin to be used as antimicrobial packaging. The developed films were further applied to minas frescal cheese, whose surface was previously inoculated with *Listeria monocytogenes*. The results of this study showed that nisin could be used as an active barrier in food contamination controlling; films with 2 g/100 g significantly reduced the bacterial count after 4 days, while films with 6 g/100 g entirely inhibited bacterial growth.

Divsalar et al. (2018) [86] developed a bilayer film with antimicrobial properties using chitosan, cellulose, and nisin. Films containing 1000 µg/mL totally inactivated *Listeria monocytogenes* at the surface of ultra-filtered (UF) cheese, after storage for 14 days at 4 °C.

Nanoparticles based on nisin-loaded poly-γ-glutamic acid/chitosan contained in PEO nanofibers were developed by Cui et al. (2017) [82] and their anti-*Listeria* activity was determined on cheese. The results of this study showed that the obtained nanofibers presented good antibacterial activity against *Listeria monocytogenes*, with no effect on the sensory attributes of tested cheese samples.

Whey protein isolate films incorporated with nisin were obtained by Seydim et al. (2020) [133] and used as layers on kasar cheese slices which were inoculated with *Listeria monocytogenes*, *Salmonella Enteritidis*, *Escherichia coli* 0157:H7, *Staphylococcus aureus*, and *Penicillium* spp. The developed films presented microbial inactivation against *Salmonella Enteritidis* and *Listeria monocytogenes*.

Berti et al. (2019) [134] studied the ripening of Gouda cheese coated with an edible coating based on tapioca starch and glycerol containing nisin and natamycin. The coating did not affect the physicochemical parameters of cheese samples or Lactobcilli development during the ripening process and presented a better barrier against external contamination compared to the control samples for the development of a mixture formed of *Listeria innocua* and *Saccharomyces cerevisiae*.

Biodegradable antimicrobial films based on HPMC (hydroxypropylmethylcellulose) incorporated with nisin Z were developed by Freitas et al. (2020) [135] and applied to sliced mozzarella cheese. It was observed that nisin presented antimicrobial activity against gram-positive bacteria, such as *Staphylococcus aureus* and *Listeria innocua*, but also inhibited gram-negative bacteria, such as *Salmonella enterica*. Furthermore, films with 10% wt. were effective in mesophilic microorganisms’ inhibition compared with the control, during 8 days of storage.

Soto et al. (2019) [136] obtained nisin-loaded amaranth protein isolate/pullulan nanofibers using the electrospinning method, evaluating its antimicrobial effectiveness on fresh cheese. The results showed complete inactivation of microorganisms after 142 h for *Salmonella Typhimurium*, 120 h for *Listeria monocytogenes,* and 170 h for *Leuconostoc mesenteroides*.

Edible films containing nisin and natamycin were used on Port Salut cheese to determine its microbiological stability by Resa et al. (2016) [137]. The developed films inhibited the growth of yeasts and molds and also controlled the growth of Port Salut cheese’s own psychrotrophic bacteria in refrigerated conditions. During the entire storage period, the films inhibited superficial contamination of a mixed culture formed of *Listeria innocua* and *Saccharomyces cerevisiae*.

As this study shows, nisin has been used in combination with packaging materials, this way being directly in contact with food products. This method can be used to incorporate bacteriocins within existing or novel packaging materials, thereby protecting food from external factors. Since the spoilage of food starts with the development of microorganisms on its surface, using bacteriocins directly in contact with food in packages leads to an improvement in the safety and quality of the product, even an extension of its shelf-life [34]. Several materials have been used as matrices for nisin incorporation (e.g., gelatin, chitosan, starch. PBAT, PHB, PLA, and PEO) to develop edible coating or packaging films with antimicrobial properties, which were further applied on different foods (meat- or dairy-based products) and the results have in common some specific outcomes, such as the shelf-life prolongation of products, maintaining physical-chemical and sensorial attributes and the inhibition of bacterial foodborne pathogens.

To summarize the above-presented information, a synthesis is presented in Table 2, where the most important results of the studied papers can be observed.

## 4. Conclusions and Future Perspectives

Presently, food preservation represents one of the major challenges in the food industry. The development of packaging materials with antimicrobial activity, but packaging that is also biodegradable, biocompatible, and environmentally friendly, could be one of the solving solutions to this challenge.

A new way for developing antimicrobial packaging is represented by nanotechnology, which was integrated into food science under different forms such as nanofibers, nanoemulsions, nanoliposomes, or nanoparticles which, in turn were integrated into innovative packaging to improve its performance (the antimicrobial and antioxidant attributes) and its impact on the environment (biodegradability and nontoxicity attributes). Nisin is often incorporated in the packaging in one of the above-stated forms, so research must be performed in order to determine the optimal solution for nisin incorporation in different matrices in order to fully exploit its attributes (its antimicrobial effect, stability, and efficacy) in terms of its quality as a preservative. Since the use of nanomaterials, incorporating nisin in the development of food packaging is still a new field and there are not many studies in the literature. As a result, data on both their migration and toxicity are currently limited.

The multitude of research studies over the last years increased the knowledge of nisin and its applications in different domains, especially in food science. This study presented the most recent results obtained in the field regarding the incorporation of nisin as an antimicrobial agent in different packaging materials, and the results obtained through some applications of such active materials on meat and dairy products in order to prolong their shelf-life. Based on this study, we can conclude that nisin is highly effective against gram-positive bacteria and can lead to shelf-life prolongation of various food products of animal origin. While nisin is less effective against gram-negative bacteria, studies show that it can be used in combination with chelators (such as EDTA) or other antimicrobials (the hurdle approach) to enhance antimicrobial activity against gram-negative bacteria. Additionally, recent research has focused on nisin applications in food packaging, such as edible films and polymeric packaging materials, and these active packaging materials can be used in the hurdle approach for food preservation. However, additional research must be performed in some related areas, despite the progress made so far. Further studies are needed for a fully understanding of the nisin mechanism of action on various microorganisms, especially on food spoilage ones, and also for clarification of the application of the antimicrobial materials on different food products, in order to extend their shelf-life and obtain a deeper clarification of the behavior of nanoparticles introduced into the food packaging when they come into contact with the food product, as well as the possible mechanisms involved in migration.

## Figures and Tables

**Table 1:** Aspects regarding packaging materials containing nisin and their antimicrobial efficiency.

Developed Packaging Materials Containing Nisin	Antimicrobial Action	References
PVA-AHSG films incorporated nisin	tested on *Listeria innocua*—inhibition zones increasing with nisin concentration in films	[79]
nisin-incorporated triaxial fibers	excellent biocidal activities for up to 5 days	[80]
BOPP/LDPE coated with NC and nisin	inhibited the growth of *Listeria monocytogenes* by 94%	[81]
PEO nanofibers containing nisin-loaded poly-g-glutamic acid/chitosan	great antimicrobial activity; effective against *Listeria monocytogenes* on cheese, with no alteration of sensorial properties	[82]
nisin grafted magnetic graphene oxide nanohybrids	long term antibacterial activity and stability (after 6 months of storage) against *Bacillus subtilis* and *Staphylococcus aureus*	[83]
chitosan-coated nisin-silica liposome	sustained antibacterial activity against *Listeria monocytogenes*; no effect on the sensory properties of cheddar cheese	[84]
starch/halloysite/nisin based films	films with 2 g/100 g nisin significantly reduced the initial bacterial load of minas frescal cheese after 4 days; films with 6 g/100 g nisin completely inhibited the development of *Listeria monocytogenes*	[85]
bilayer film based on chitosan, cellulose, and nisin (500 and 1000 g/mL)	films with great antimicrobial properties; the one containing 1000 g/mL inhibited completely the development of *Listeria monocytogenes* on the surface of ultra-filter (UF) white cheese after storage at 4 °C for 14 days	[86]
nisin-incorporated cellulose films	presented antimicrobial activity against *Staphylococcus aureus*, *Listeria monocytogenes*, *Alicyclobacillus acidoterrestris,* and *Bacillus cereus*	[87]
nisin and ε-PL with chitosan coating	decreased the growth of yeast and mold, total viable counts, total coliforms count, *Staphylococcus aureus,* and *Pseudomonas* spp. in fresh-cut carrots	[88]
N-succinyl chitosan-based films with nisin and Perilla essential oil	good antioxidant and antibacterial activity against *Staphylococcus aureus*, *Escherichia coli*, *Salmonella enteritidis,* and *Pseudomonas tolaasii*	[89]
novel films with chitosan/nano/SiO_2_/nisin films	used as coatings for cantaloupes—reduced the yeast and mold counts	[90]
chitosan coating plus SiO_2_ nanoparticles and nisin	the coating containing nisin inhibited the microbial populations for molds/yeast and mesophilics on blueberries	[91]
chitosan/silica nanoparticle/nisin films	coated blueberries with chitosan/silica nanoparticle/nisin films reported the lowest microbial contamination counts	[92]
nisin in polylactide and polylactide/PEG blends	good antimicrobial activity against *Micrococcus luteus*	[93]
starch/PBAT films containing nisin (2%) and ε-PL (1%)	efficiently inhibited the development of *Staphylococcus aureus* and *Escherichia coli*	[94]
PBAT incorporated with nisin	inhibitory effect on *Listeria monocytogenes*	[95]
nisin anchored on oxidized cellulose film	good antimicrobial activity against *Alicyclobacillus acidoterrestris* DSM 3922T	[96]
incorporated nisin and ε-polylysine into corn starch phosphate/nanocrystalline cellulose-based films	antimicrobial activity against gram-positive bacteria (*Staphylococcus aureus*) and gram-negative bacteria (*Escherichia coli*)	[97]
nisin loaded chi-tosan/PVA film	the concentration of *Staphylococcus aureus* decreased from 100% to 11.65% when nisin content increased from 0 to 10%	[98]
nisin-incorporated chitosan films	effective against *Listeria monocytogenes*	[99]
nisin-containing bacterial cellulose film	great inhibitory effect on gram-positive bacteria (*Staphylococcus aureus* ATCC 6538)	[100]
encapsulated nisin in silica	great antibacterial activity against both gram-positive and gram-negative bacteria, especially against *Escherichia coli*	[101]
chitosan-PLA composite film with nisin and EDTA	high antibacterial activity	[52]
starch/PVA/nisin films	Films containing nisin showed an inhibitory effect against *Staphylococcus aureus*, *Pseudomonas aeruginosa*, *Salmonella Choleraesuis,* and *Escherichia coli*	[102]

PVA—poly(vinyl alcohol); AHSG—Alyssum homolocarpum seed gum; BOPP—biaxially oriented poly(propylene); LDPE—low-density polyethylene; PEO—poly(ethylene oxide); ε-PL—ε-poly(lysine); PEG—poly(ethylene glycol); NC—nanofibrillated cellulose; PBAT—butylene adipate-co-terephthalate; PLA—poly(lactic acid); EDTA—ethylenediaminetetraacetic acid.

**Table 2:** Effects of different materials containing nisin on products of animal origin quality.

Product	Material	Results	References
Meat and meat products			
minced pork meat	gelatin-based films incorporated with nisin and catechin	retarded lipid oxidation and microbial growth	[115]
fresh pork loin	coatings based on nisin/gallic acid/chitosan	preserved product color, had lower lipid and protein oxidation, the lowest TBARS value, and presented a synergistic bactericidal effect	[116]
pork meat	nisin, EDTA, and PBAT/TPS blends	inhibited lipid degradation, microbial growth, stabilized meat redness, delayed discoloration	[117]
Yao meat	nanoemulsion incorporated with nisin, star anise essential oil, and polylysine	no effect on sample moisture content for 20 days, maintaining good color and odor, the extension of shelf-life was from 8 to 16 days	[118]
sliced ham	Poly(hidroxybutyrate) (PHB)/poly(caprolactone) (PCL)/nisin films	effective against *Lactobacillus plantarum* CRL691, prolonged shelf-life	[108]
dry-cured Iberian ham	Chitosan-based films containing nisin and/or rice bran in combination with high-pressure processing (HPP)	reduced the population of L. monocytogenes by 6 log CFU/g	[53]
ready-to-eat ham	cellulose nanofibrils/nisin nanohybrid films	complete inhibition of *Listeria monocytogenes* during storage for 7 days at 4 °C	[119]
Vienna sausage	collagen casting impregnated with nisin	extended shelf-life for at least 90 days at 4 °C and 49 days at 10 °C	[120]
raw turkey breast slices	pullulan films containing lauric arginate and nisin Z	reduced *Salmonella typhimurium*, *Salmonella enteritidis Staphylococcus au-reus* and *Listeria monocytogenes* Scott A load during cold storage	[121]
turkey bologna	coatings containing nisin applied on two types of materials used as substrates	inhibition of *Listeria monocytogenes* during the entire period of testing	[77]
minced beef	combinations of lysozyme 500 U/mL (L), nisin 1000 IU/mL (N), EDTA nanoparticles 5 mM (E), and ZnO nanoparticles 12 mM (Z)	the synergistic antimicrobial effect observed for LNZ and LNEZ, which efficiently inhibited *Escherichia coli* O157:H7, *Listeria monocytogenes,* and *Bacillus cereus* during storage al 4 °C for 15 days	[122]
beef	nanocoatings based on bacterial cellulose nanocrystals and nisin-loaded bacterial cellulose nanocrystals	no alteration of sensorial and physicochemical properties, reduced bacterial growth	[123]
yellow croaker	chitosan combined with nisin	nisin’s presence led to better quality parameters, controlling total viable count growth, and maintaining color and sensory acceptability	[124]
seer fish steaks	the coating formed from gel dispersion of seer fish incorporated with nisin combined with gamma irradiation	shelf-life prolonging from 7 days to 34–42 days during cold storage	[125]
fish fillets	nisin and curcumin nanomats	acceptable sensory attributes by the tenth day of storage compared to control, shelf-life extended to 12 days	[126]
fish fillets	nisin-loaded PVA-based nanofibers with polyethylene	delayed TMB and LAB development by 31% and 38%, respectively, better sensorial attributes compared to control samples	[127]
fish balls	zein incorporated nisin (54.4 AU/cm^2^) or nisin/EDTA 568 µg/cm^2^	The microbial count of *Escherichia coli*, *Enterobacter aerogenes,* and *Citrobacter freundii* decreased from 3.19 ± 0.03 log CFU/g for uncoated fish balls to less than 1 log unit for coated food products; the control recorded a microbial count of 6.08 ± 0.23 log CFU/g	[64]
bigeye snapper and tiger prawn slices	thermoplastic starch/PBAT film coated with gelatin with lauric arginate alone or in combination with nisin	in combination with nisin, reduced *Salmonella Typhimurium* ATCC 14,028 and *Vibrio parahaemolyticus* after 28 days at 4 °C and *Salmonella Typhimurium* after 60 days at −20 °C; reduced *Vibrio parahaemolyticus* on frozen samples after 14 days (bigeye snapper) and 21 days (tiger prawn)	[128]
*Penaeus vannamei* shrimp	chitosan/sodium alginate/nisin preservatives	Lower pH, TVB-N, TVC, and freeness values, decreased the predominant microbial load significantly, longer shelf-life	[129]
fish fillets	chitosan-PLA composite added with nisin and EDTA	significant reduction in coliform, mesophile, and spoilage bacteria loads and of the total volatile basic nitrogen content during storage at 25 °C and 4 °C	[52]
Dairy products			
minas frescal cheese	nanocomposite films based on starch/halloysite/nisin	films with 2 g/100 g nisin significantly reduced the bacterial count after 4 days, while films with 6 g/100 g nisin entirely inhibited bacterial growth	[85]
UF cheese	bilayer film based on chitosan, cellulose, and nisin	films containing 1000 µg/mL totally inactivated *Listeria monocytogenes* after storage for 14 days at 4 °C	[86]
cheese	nanoparticles based on nisin-loaded poly-γ-glutamic acid/chitosan contained in PEO nanofibers	good antibacterial activity against *Listeria monocytogenes*, no effect on the sensory attributes	[82]
kasar cheese slices	whey protein isolate films incorporated with nisin	microbial inactivation against *Salmonella Enteritidis* and *Listeria monocytogenes*	[133]
Gouda cheese	an edible coating based on tapioca starch and glycerol, containing nisin and natamycin	no effect on physicochemical parameters during the ripening process, a better barrier against external contamination	[134]
sliced mozzarella cheese	films based on hydroxypropylmethylcellulose (HPMC) incorporated with nisin Z	nisin presented antimicrobial activity *Staphylococcus aureus*, *Listeria innocua*, and *Salmonella enterica*; films with 10% wt. were effective in mesophilic microorganisms’ inhibition during 8 days of storage	[135]
fresh cheese	Nisin-loaded amaranth protein isolate/pullulan nanofibers	complete inactivation of microorganisms after 142 h for *Salmonella Typhimurium*, 120 h for *Listeria monocytogenes,* and 170 h for *Leuconostoc mesenteroides*	[136]
Port Salut cheese	edible films containing nisin and natamycin	inhibited the growth of yeasts and molds, controlled the growth of product’s own psychrotrophic bacteria, inhibited superficial contamination of a mixed culture of *Listeria innocua* and *Saccharomyces cerevisiae*	[137]
